# Genetic variation of hypoxia tolerance in farmed fish: a systematic review for selective breeding purposes

**DOI:** 10.1186/s12711-026-01032-1

**Published:** 2026-02-06

**Authors:** Sergio P. Barahona, Nicolás Salinas-Parra, Rodrigo Pulgar, José Gallardo-Matus

**Affiliations:** 1 Laboratorio de Genética y Genómica Aplicada, Escuela de Ciencias del Mar, Pontificia Universidad Católica de Valparaíso, Avenida Universidad 330, 2373223 Valparaíso, Chile; 2 Laboratorio de Genética y Genómica e Interacciones Biológicas, Instituto de Nutrición y Tecnología de los Alimentos (INTA), Universidad de Chile, El Líbano 5524, 7830490 Santiago, Chile

## Abstract

**Background:**

Accelerating climate change has intensified hypoxic events in aquatic ecosystems. In aquaculture, high stocking densities make farmed fish particularly vulnerable to these episodes, leading to negative economic repercussions. This has driven interest in selective breeding for hypoxia tolerant fish as a potential mitigation strategy. In this context, the present systematic review synthesizes and critically evaluates current knowledge on genetic variation associated with hypoxia tolerance in farmed fish species. A literature search was conducted in Scopus and Web of Science following the Preferred Reporting Items for Systematic reviews and Meta-Analyses 2020 guidelines.

**Results:**

In total, 964 articles were identified, of which 41 met the inclusion criteria, encompassing 26 species and three hybrid lines. Among the farmed fish, the blunt snout bream, rainbow trout, common carp, and Nile tilapia were the most extensively studied. The most commonly used metrics to assess hypoxia tolerance included: (1) time or oxygen level at which loss of equilibrium occurs, (2) survival time or status (alive or dead), and (3) critical oxygen partial tension measured via respirometry. Substantial phenotypic variability in hypoxia tolerance across families, strains, gynogenetic lines, growth transgenic lines, hybrid lines, and species was reported in most studies. Although single nucleotide polymorphism associated with hypoxia tolerance were identified in several studies, heritability estimates were reported in only three of these, ranging from 0.28 to 0.65, underscoring the need for further research to strengthen the basis for selective breeding applications. Furthermore, candidate genes identified across studies were involved in a wide range of biological processes underlying hypoxia responses, including hypoxia signaling and its regulation (HIF-related genes and their inhibitors, such as *HIF1αn*), angiogenesis, energy metabolism, oxidative stress defense, erythropoiesis, ion regulation, DNA repair, and immune and apoptotic processes.

**Conclusions:**

As aquatic environments become more hypoxic, the findings of this review underscore the potential of the inherent genetic diversity for hypoxia tolerance present in farmed fish populations. In this context, genomic selection and gene editing emerge as promising tools for developing hypoxia tolerant fish lines. Further research under production conditions is essential before implementation of these approaches in practical in breeding programs, particularly to evaluate the most appropriate trait(s) for measuring hypoxia tolerance, potential correlated responses with other economically important traits, and the overall feasibility at an industry scale.

## Background

Hypoxia is a complex environmental stressor for aquatic organisms, defined by reduced levels of dissolved oxygen in water [1–3]. Because dissolved oxygen limits aerobic metabolism in aquatic organisms [4], its depletion severely reduces the yield, quality, and survival of farmed fish [5], thereby threatening the sustainability of aquaculture worldwide. In aquaculture systems, hypoxia arises from multiple factors that can be classified as environmental or production driven.

Major environmental drivers of hypoxia events include climate change [3, 6] and thermal stratification caused by diurnal and seasonal temperature fluctuations [7–9]. Additional contributors that accelerate oxygen depletion in aquatic systems include upwelling of oxygen depleted deep waters [10, 11], eutrophication [1, 12], and microbial decomposition of harmful algal blooms [13–15]. Furthermore, because the gills of fish are directly exposed to the surrounding environment, they are highly vulnerable to damage caused by various biotic agents (e.g., jellyfish, amoebae) [16–20], which can further impair the ability of farmed fish to tolerate hypoxic conditions.

In many cases, factors related to production systems or deficiencies within them can intensify oxygen depletion. High stocking densities, excessive feeding, waste accumulation, and biofouling of cage nets [21, 22], can all lead to sudden drops in oxygen levels that are difficult to predict [8, 23]. Because farmed fish, particularly in marine systems, cannot escape hypoxia, individuals with low tolerance often die. Mass mortality events triggered by hypoxia have been reported in various production systems worldwide [24–26], highlighting the severity of this stressor [27, 28]. Oxygenation systems are commonly employed in aquaculture to counteract the effects of high stocking densities, and the same technologies, including nanobubble diffusion systems, water jets, paddles, air injection [29–31], or artificial upwelling [32], are also used to mitigate hypoxia events. However, these technologies can be costly to implement and maintain on a large scale [33] and may fail or are susceptible to operator error, which, although infrequent, can lead to severe mortality events [34].

### Physiological and tissue-level effects of hypoxia

Hypoxic events do not always lead to fish mortality, and their impact depends on whether hypoxia is acute (short-term and extreme) or chronic (prolonged exposure). In some cases, hypoxia induces sublethal effects that alter physiological, tissue, and molecular processes [3]. At the physiological level, hypoxia reduces metabolic rate, growth rate, feed conversion efficiency, and immune response [35–38]. These adverse effects increase susceptibility to pathogens and reduce the capacity of fish to adapt to environmental change, ultimately compromising survival [39]. At the tissue level, hypoxia can cause gill damage [40], while chronic hypoxia can induce oxidative stress, leading to DNA damage, increased lipid peroxidation, and persistent activation of DNA repair pathways, even after the restoration of normoxic conditions [41]. However, fish have evolved physiological and metabolic mechanisms to cope with hypoxia [42]. For instance, they can enhance O_2_ uptake and transport, upregulate anaerobic ATP production pathways such as glycolysis, and suppress molecular processes with high ATP demand such as protein synthesis [4, 36, 43, 44]. Most of these mechanisms are mediated by hypoxia-responsive genes, which are activated by specific pathways. The HIF-1 pathway, composed of HIF-1α and HIF-1β, controls the expression of multiple hypoxia-sensitive genes such as *VEGF*, *EPO*, *LDH*, and *GLUT* [44–47]. Additional signaling pathways, including MAPK (Mitogen-Activated Protein Kinase) and PI3K/AKT/mTOR, play key roles in cardiac tolerance to hypoxia and in the regulation of angiogenic responses under hypoxic conditions in fish, respectively [48, 49]. Since fish exhibit varying degrees of hypoxia tolerance [50, 51], even within the same species [52], understanding the genetic architecture of this trait could inform artificial selection strategies to enhance resilience of aquaculture systems to hypoxia [53].

### Phenotyping strategies used in hypoxia tolerance testing

A simple and cost-effective method for assessing hypoxia tolerance in fish is the loss-of-equilibrium (LOE) test [54]. This protocol consists of gradually reducing oxygen levels by injecting nitrogen and recording the time at which the fish loses its ability to maintain dorsoventral balance. A short time to LOE (t_LOE_) indicates a hypoxia-sensitive individual, whereas a longer t_LOE_ denotes greater hypoxia tolerance [52, 54, 55]. Additional LOE-based metrics include the oxygen concentration (mg/L, %), or partial pressure (kPa or torr) at which equilibrium loss occurs (LOE_crit_), which quantifies the oxygen concentration (mg/L), saturation (%), or partial tension (kPa or torr) at which equilibrium loss occurs [53, 56], and time or oxygen concentration at which 50% of fish lose equilibrium (LOE_50_), which represents the time or oxygen concentration (mg/L) at which 50% of fish in a group lose equilibrium [51, 57]. Beyond LOE-based assessments, hypoxia tolerance can be evaluated using critical oxygen partial tension (P_crit_), which reflects the ability of fish to uptake dissolved oxygen from water [58–60], incipient lethal oxygen saturation (ILOS) [61, 62], survival time [63], and binary survival or mortality traits [64]. Additional physiological and molecular indicators of hypoxia tolerance include cardiac function parameters [52, 65], gill remodeling [53], metabolic activity [66], enzymatic activities [67], differential gene expression [46], and transcriptomic analysis [5, 68].

### Genetic variation and heritability of hypoxia tolerance

Heritability estimates for hypoxia tolerance have been reported in several commercially important fish species, although they are based on different phenotypic definitions and measurement protocols. Reported values range from moderate in rainbow trout (*Oncorhynchus mykiss*, heritability of time to loss of equilibrium = 0.28) [65], to high in large yellow croaker (*Larimichthys crocea*, heritability of time to loss of equilibrium = 0.62; heritability of survival status = 0.65) [69]. The observed genetic variability in hypoxia tolerance highlights the capacity of fish populations to adapt to expanding hypoxic environments. Understanding this genetic variation is key to designing breeding strategies that make farmed species more resilient.

At the molecular level, genetic variation associated with hypoxia tolerance has been documented at the SNP marker level, with specific SNPs and candidate genes identified in rainbow trout [65] and channel catfish [70]. SNPs located in regulatory regions, such as hypoxia-responsive elements (HREs) within promoters of HIF-target genes and in introns, enhancers, Untranslated Regions (UTR), or exons, can directly or indirectly influence gene transcription or expression efficiency, thereby modulating the hypoxia tolerance phenotype [70].

### Review objectives

Based on the above, it is clear that hypoxia tolerance could play a critical role in aquaculture sustainability, as it supports improved survival rates, reduces dependence on oxygenation systems, enhances resource efficiency, and increases resilience to climate change. Against this background, this systematic review consolidates and critically evaluates current knowledge on the genetic basis of hypoxia tolerance in farmed fish. It synthesizes advances ranging from phenotypic assessments to the identification of molecular markers, highlighting their potential applications in selective breeding programs to enhance resilience to low-oxygen environments.

## Methods

This systematic review adhered to the guidelines of the PRISMA 2020 statement [71]. A systematic search for relevant articles was conducted in the Web of Science (WoS) and Scopus databases in September 2024, using the following search strategy (Table 1): (a) Genetic components: articles indexed in databases were included if they contained data on (1) heritability (h^2^), (2) QTL (Quantitative Trait loci), (3) SNPs, (4) genotype by environment interaction (G × E) analysis, (5) genome-wide association studies (GWAS), (6) candidate genes, (7) phenotypic variability among families, strains, populations, or species, or (8) genetic correlations; (b) Hypoxia-related terms: articles were included if they contained terms such as (1) tolerance to hypoxia, (2) oxygen stress, (3) oxygen variability, (4) hypoxic stress, (5) oxygen metabolism, (6) LOE, etc.; and (c) target organism: the third block of the search strategy included the common names of the top 15 inland and top 15 marine or coastal fish species with the highest global production according to the Food and Agriculture Organization (FAO) 2024 report [72]. All farmed fish species identified through the search strategy were included, regardless of their explicit mention in the FAO 2024 report.

**Table 1:** Summary of boolean keywords used in the database search

Search block	Main keywords and operators
Genetic terms	“genetic variation” or “genetic variant” or “genetic variability” or “genetic architecture” or “geographic variation” or genotype* or allele* or polymorphism or “genomic variant*” or “phenotypic plasticity” or “phenotypic variation” or strain* or inter-strain* or inter-population* or “population differences” or line* or family* or inter-family* or correlation or “genetic correlation” or GWAS or “genome-wide association study” or “genome-wide association analysis” or QTL* or “genomic prediction” or “single nucleotide polymorphism” or “candidate SNPs” or “novel SNP*” or “SNP chip” or SNP* or “SNP array” or “SNP panel*” or marker* or “genomic selection” or GS or “candidate genes” or “annotated candidate genes” or imput* or heritability* or “putative candidate genes” or “genetic improvement” or “artificial selection” or “selective breeding”
Hypoxia tolerance terms	“hypoxia tolerance” or “tolerance to hypoxia” or “oxygen stress” or “oxygen tolerance” or “oxygen variability” or “hypoxic stress” or “hypoxic conditions” or “low oxygen tolerance” or “hypoxia-resilient” or “oxygen metabolism” or “hypoxia tolerance trait*” or LOE or “loss of equilibrium”
Fish terms	fish* or finfish* or “teleost fish*” farmed or “farmed fish” or aquaculture or fisher* or inland or marine or “grass carp” or “silver carp” or “Nile tilapia” or “common carp” or catla or “bighead carp” or Carassius or “striped catfish” or “roho labeo” or “clarias catfish*” or “tilapias nei” or “Wuchang bream” or “rainbow trout” or “black carp” or “largemouth black bass” or “Atlantic salmon” or “milkfish” or “mullets nei” or “githead seabream” or “large yellow croaker” or “European seabass” or “groupers nei” or “coho salmon” or “Japanese seabass” or “giant seaperch” or “red drum”
Combined Boolean structure	(Genetic terms) AND (Hypoxia tolerance terms) AND (Fish terms)

Articles focusing exclusively on other traits, such as thermal tolerance, were excluded to maintain a strict focus on genetic variation associated with hypoxia tolerance, although these traits may be physiologically related. The following types of publications were also excluded: (1) graduate theses, editorials, letters to the editor, reviews, book chapters, conference proceedings, and abstracts; (2) non-English language manuscripts; (3) articles without full-text availability; (4) studies focusing exclusively on transcriptomics, gene expression, biomarkers, and or epigenetic markers were excluded because the review specifically aimed to identify heritable genetic variation—while transcriptomic or epigenetic studies can reveal functional responses to hypoxia, they do not provide estimates of genetic variability or heritability that are directly applicable to selective breeding programs; (5) studies on species other than fish or on fish not farmed; (6) studies that did not compare tolerance to hypoxia across groups (e.g., gynogenetic lines or strains); (7) studies analyzing growth rate, immunity, biochemical parameters, and or physiological responses under varying hypoxic or anoxic conditions or hypoxia-induced stress; and (8) studies examining the effects of diets or pharmaceuticals on hypoxia tolerance.

The Endnote software package was used to manage retrieved references, to automatically remove duplicates, and to organize sources by theme. Titles and abstracts were screened against predefined inclusion and exclusion criteria. Records deemed potentially relevant were subsequently assessed through full-text evaluation for eligibility. One reviewer conducted the initial screening, and a second reviewer independently verified the eligibility decisions. Any discrepancies in inclusion or exclusion were resolved through consensus. While this approach does not replace a formal risk of bias assessment, it helps to minimize selection bias and promotes consistency and scientific rigor in the study selection process.

## Results and discussions

Given the integrative nature of the available evidence, the main findings are presented and discussed together to facilitate interpretation of patterns emerging across studies, species, and methodological approaches. We first provide a concise synthesis of the key results derived from the study selection and data extraction process (Tables 2, 3, 4), and then examine in greater depth the following themes: (1) the methods and traits used to assess hypoxia tolerance and their suitability for breeding programs, (2) genetic correlations between hypoxia tolerance and other economically relevant traits, including growth, disease resistance, and thermal tolerance, (3) sources of genetic variation across strains, populations, ploidy levels, gynogenetic lines, and hybrids, (4) insights from modern genomic approaches, including GWAS, QTL mapping, genomic selection, and gene editing, and (5) outcomes of selective breeding efforts. Together, these findings provide a framework for evaluating the feasibility and limitations of incorporating hypoxia tolerance into selective breeding strategies in farmed fish under increasingly variable and hypoxic aquaculture environments.

**Table 2:** Overview of the phenotypic studies of hypoxia tolerance identified in this review (*n* = 25)

Species	Method	Type of test	Time to exposure to hypoxia	Trait (s)	Number of groups compared	Types of groups compared	Reference
Rainbow trout (*Oncorhynchus mykiss*)	Hypoxia challenge test	Acute	~ 2.5 h	t_LOE_ (min)	3	Genetically identical lines with different growth rates	[55]
Rainbow trout (*Oncorhynchus mykiss*)	Hypoxia challenge test	Acute	~ 3 h	t_LOE_ (min)	2	Strains with different growth rates	[66]
Rainbow trout (*Oncorhynchus mykiss*)	Hypoxia challenge test, Respirometry	Acute	NA	ILOS (% O_2_ Saturation), P_crit_	3	Strains	[61]
Nile tilapia (*Oreochromis niloticus*)	Hypoxia challenge test	Acute	NA	DO (mg/L) at different ASR thresholds	2	Strains	[73]
Nile tilapia (*Oreochromis niloticus*)	Hypoxia challenge test	Acute	NA	pO_2_ (kPa) at different ASR thresholds	5	Random size-matched individuals	[74]
Atlantic salmon (*Salmo salar*)	Hypoxia challenge test	Acute	2 h	t_LOE_ (min)	41	Families	[52]
Atlantic salmon (*Salmo salar*)	Mortality hypoxia test	Acute	NA	Survival of embryos (Number)	4	Wild genetically different populations from different rivers	[75]
Barramundi (*Lates calcarifer*)	Respirometry	Acute	6 h	P_crit_ (% air sat)	2	Two populations geographically separated	[76]
Channel catfish (*Ictalurus punctatus*)	Hypoxia challenge test	Acute	~ 1.6 h	t_LOE_ (min)	6	Strains	[77]
Blunt snout bream (*Megalobrama amblycephala*)	Hypoxia challenge test	Acute	NA	LOE_crit_ (mg/L)	2	Control strain versus genetically improved strain	[53]
Blunt snout bream (*Megalobrama amblycephala*)	Hypoxia challenge test	Acute	NA	LOE_crit_ (mg/L)	2	Control strain versus genetically improved strain	[78]
Blunt snout bream (*Megalobrama amblycephala*)	Hypoxia challenge test	Acute	4 h	LOE_crit_ (mg/L), Average number of fish as they began to lose equilibrium	2	Control versus hybrid	[79]
Hybrid Blunt snout bream (*Megalobrama amblycephala* × *Culter alburnus*)	Hypoxia challenge test	Acute	4 h	LOE_crit_ (mg/L), Average number of fish as they began to lose equilibrium	2	Control versus hybrid	[79]
Qing bo/Hiina astelparrak (*Spinibarbus sinensis*)	Respirometry	Acute	3 h	P_crit_ (torr)	6	Species	[80]
Chinese bream (*Parabramis pekinensis*)	Respirometry	Acute	3 h	P_crit_ (torr)	6	Species	[80]
Grass carp (*Ctenopharyngodon idellus*)	Respirometry	Acute	3 h	P_crit_ (torr)	6	Species	[80]
Big head carp (*Aristichthys nobilis*)	Respirometry	Acute	3 h	P_crit_ (torr)	6	Species	[80]
Silver carp (*Hypophthalmichthys molitrix*)	Respirometry	Acute	3 h	Pcrit (torr)	6	Species	[80]
Common carp (*Cyprinus carpio var Jian*)	Respirometry	Acute	3 h	P_crit_ (torr)	6	Species	[80]
Qing bo/Hiina astelparrak (*Spinibarbus sinensis*)	Hypoxia challenge test, Respirometry	Acute	NA	LOE_crit_ (kPa), P_crit_ (kPa)	8	Species	[50]
Common carp (*Cyprinus carpio*)	Hypoxia challenge test, Respirometry	Acute	NA	LOE_crit_ (kPa), P_crit_ (kPa)	8	Species	[50]
Crucian carp (*Carassius carassius*)	Hypoxia challenge test, Respirometry	Acute	NA	LOE_crit_ (kPa), P_crit_ (kPa)	8	Species	[50]
Thick-jawed bream (*Megalobrama pellegrini*)	Hypoxia challenge test, Respirometry	Acute	NA	LOE_crit_ (kPa), P_crit_ (kPa)	8	Species	[50]
Silver carp (*Hypophthalmichthys molitrix*)	Hypoxia challenge test, Respirometry	Acute	NA	LOE_crit_ (kPa), P_crit_ (kPa)	8	Species	[50]
Bighead carp (*Aristichthys nobilis*)	Hypoxia challenge test, Respirometry	Acute	NA	LOE_crit_ (kPa), P_crit_ (kPa)	8	Species	[50]
Grass carp (*Ctenopharyngodon idellus*)	Hypoxia challenge test, Respirometry	Acute	NA	LOE_crit_ (kPa), P_crit_ (kPa)	8	Species	[50]
Black carp (*Mylopharyngodon piceus*)	Hypoxia challenge test, Respirometry	Acute	NA	LOE_crit_ (kPa), P_crit_ (kPa)	8	Species	[50]
Sablefish (*Anoplopoma fimbria*)	Hypoxia challenge test, Respirometry	Acute	~ 1.7 h	LOE_crit_ (% air sat), t_LOE_ (min), P_crit_ (% air sat)	2	Juvenile versus adult	[81]
Mountain carp (*Schizothorax prenanti*)	Hypoxia challenge test, Respirometry	Acute	1 h	LOE_50_ (mg O_2_L^−1^), P_crit_ (mg O_2_L^−1^), ASR_50_	11	Species	[51]
Sharp-jaw barbell (*Onychostoma sima*)	Hypoxia challenge test, Respirometry	Acute	1 h	LOE_50_ (mg O_2_L^−1^), P_crit_ (mg O_2_L^−1^), ASR_50_	11	Species	[51]
Qing bo/Hiina astelparrak (*Spinibarbus sinensis*)	Hypoxia challenge test, Respirometry	Acute	1 h	LOE_50_ (mg O_2_L^−1^), P_crit_ (mg O_2_L^−1^), ASR_50_	11	Species	[51]
Crucian carp (*Carassius carassius*)	Hypoxia challenge test, Respirometry	Acute	1 h	LOE_50_ (mg O_2_L^−1^), P_crit_ (mg O_2_L^−1^), ASR_50_	11	Species	[51]
Common carp (*Cyprinus carpio*)	Hypoxia challenge test, Respirometry	Acute	1 h	LOE_50_ (mg O_2_L^−1^), P_crit_ (mg O_2_L^−1^), ASR_50_	11	Species	[51]
Bighead carp (*Aristichthys nobilis*)	Hypoxia challenge test, Respirometry	Acute	1 h	LOE_50_ (mg O_2_L^−1^), P_crit_ (mg O_2_L^−1^), ASR_50_	11	Species	[51]
Silver carp (*Hypophthalmichthys molitrix*)	Hypoxia challenge test, Respirometry	Acute	1 h	LOE_50_ (mg O_2_L^−1^), P_crit_ (mg O_2_L^−1^), ASR_50_	11	Species	[51]
Chinese bream (*Parabramis pekinensis*)	Hypoxia challenge test, Respirometry	Acute	1 h	LOE_50_ (mg O_2_L^−1^), P_crit_ (mg O_2_L^−1^), ASR_50_	11	Species	[51]
Grass carp (*Ctenopharyngodon idellus*)	Hypoxia challenge test, Respirometry	Acute	1 h	LOE_50_ (mg O_2_L^−1^), P_crit_ (mg O_2_L^−1^), ASR_50_	11	Species	[51]
Black carp (*Mylopharyngodon piceus*)	Hypoxia challenge test, Respirometry	Acute	1 h	LOE_50_ (mg O_2_L^−1^), P_crit_ (mg O_2_L^−1^), ASR_50_	11	Species	[51]
Chinese hook snout carp (*Zacco platypus*)	Hypoxia challenge test, Respirometry	Acute	1 h	LOE_50_ (mg O_2_L^−1^), P_crit_ (mg O_2_L^−1^), ASR_50_	11	Species	[51]
Rainbow trout (*Oncorhynchus mykiss*)	Hypoxia challenge test	Acute	~ 7 h	t_LOE_ (min)	3	Triploid and diploid strains	[82]
Rainbow trout (*Oncorhynchus mykiss*)	Hypoxia challenge test	Acute	3 h	t_LOE_ (min), LOE_crit_ (kPa)	2	Triploid and diploid strains	[83]
Brook trout (*Salvelinus fontinalis*)	Hypoxia challenge test	Acute	~ 2.6 h	t_LOE_ (min), LOEcrit (kPa)	2	Triploid and diploid strains	[84]
Blunt snout bream (*Megalobrama amblycephala*)	Hypoxia challenge test	Acute	4 h	LOE_crit_ (mg/L)	3	Gynogenetic lines	[85]
Blunt snout bream (*Megalobrama amblycephala*)	Hypoxia challenge test	Acute	NA	LOE_crit_ (mg/L)	2	Gynogenetic lines	[86]
Common carp (*Cyprinus carpio*)	Mortality hypoxia test	Acute	~ 7 h	Survival time (min), Survival status (alive/dead)	8	Families (Growth hormone (GH)-transgenic common carp v/s Control)	[87]
Rainbow trout (*Oncorhynchus mykiss*)	Hypoxia challenge test, Mortality hypoxia test	Acute	NA	Initial loss of equilibrium (mg/L), final loss of equilibrium (mg/L), and mortality.	4	Parasite resistant strains	[88]
Hybrid catfish (*Pelteobagrus fulvidraco × Leiocassis longirostris*)	Hypoxia challenge test	Acute	18 h	Floating head 50 (mg/L)	5	Hybrid types	[89]
Hybrid catfish (*Ictalurus punctatus × Ictalurus furcatus*)	Hypoxia challenge test	Acute	NA	Percentage of non-tolerant fish (%)	4	Four genetic groups (crosses)	[90]

**Table 3:** General information concerning hypoxia tolerance from genetic and genomic articles identified in this review (*n* = 16)

Specie	Method	Type of test	Time to exposure to hypoxia	Trait (s)	Type of genetics study	Genotyping method	SNPs	Fish	QTLs	PVE	h^2^	Reference
Nile tilapia (*Oreochromis niloticus*)	Hypoxia challenge test	Acute	NA	t_LOE_ (s)*	SNP association studies	Sanger sequencing	5	192	1^b^	NA	NA	[91]
Barramundi (*Lates calcarifer*)	Mortality hypoxia test	Acute	NA	Survival status (alive/dead)	SNP association studies	Sanger sequencing	3	280	1^a^	NA	NA	[92]
Blunt snout bream (*Megalobrama amblycephala*)	Hypoxia challenge test	Acute	NA	LOE_crit_ (mg/L)	haplotype association study	Sanger Sequencing	2	100	1 haplotype	NA	NA	[67]
Blunt snout bream (*Megalobrama amblycephala*)	Hypoxia challenge test	Acute	NA	LOE_crit_ (mg/L)	Diplotype association study	Sanger Sequencing	2	90	1 haplotype	NA	NA	[93]
Channel catfish (*Ictalurus punctatus*)	Hypoxia challenge test	Acute	1.8 h	t_LOE_ (min)	GWAS	250 K SNP array	176,798	376	Across strains: 1^a^ and 16^b;^ within strains: Kansas (26^a^), Kmix (4^a^), Thompson (1^a^)	Across strains: 5.71%, Within strains: Kansas (25.32%), Kmix (23.04%), Thompson (32.04%)	NA	[70]
Hybrid catfish (*Ictalurus punctatus × Ictalurus furcatus*)	Hypoxia challenge test	Acute	NA	t_LOE_ (min)	GWAS	250 K SNP array	208,598	208	9^a^ y 31^b^	12.44%	NA	[49]
Large yellow croaker (*Larimichthys crocea*)	Mortality hypoxia test	Chronic	~ 33 h	Survival time (min), Survival status (alive/dead)	GWAS	ddRAD-Seq	54,224	398	Survival time: 2^a*^, Survival status: 4^a*^	Survival time: 18.04%, Survival status: 8.49%	Survival time: 0.61, Binary trait: 0.65	[64]
Large yellow croaker (*Larimichthys crocea*)	Mortality hypoxia test	Chronic	~ 33 h	Survival time (min), Survival status (alive/dead)	GWAS	55 K SNP array	120,815	372	Survival time: 5^b^, Survival status: 2^b^	Survival time: 4.98%, Survival status: 5.37%	NA	[5]
Rainbow trout (*Oncorhynchus mykiss*)	Hypoxia challenge test	Acute	5 h	t_LOE_ (min)	GWAS and GS	Imputed 665k SNP genotypes.	418,925	1297	7^a^ and 3^b^	NA	0.28	[65]
Pompano (*Trachinotus ovatus*)	Mortality hypoxia test	Acute	~ 8 h	Survival time (min)	GWAS	Whole-genome resequencing	706,991	100	4^b^	32.1%	NA	[63]
Nile tilapia (*Oreochromis niloticus*)	Mortality hypoxia test	Acute	~ 9 h	Survival time (min)	QTL mapping	ddRAD-Seq	924	96	2^a^	NA	NA	[91]
Catfish (*Pelteobagrus vachelli*)	Hypoxia challenge test	Acute	2.6 h	t_LOE_ (min)	QTL mapping	ddRAD-Seq	5,059	200	1 QTL	11.3%	NA	[94]
Silver sillago (*Sillago sihama*)	Hypoxia challenge test	Acute	~ 9 h	Time until gasping (min)	QTL mapping analysis	GBS (Genotyping by sequencing) method and Sanger Sequencing	GBS: 4,735 SNP, Sanger: 13 SNP	162	6 QTL, 5 SNP	QTL: 7.17%	NA	[95]
Large yellow croaker (*Larimichthys crocea*)	Mortality hypoxia test	Chronic	40 h	Survival time (min), Survival status (alive/dead)	GS	55 K SNP array	38,472	753	NA	NA	Survival time: 0.62 ± 0.05, Survival status: 0.65 ± 0.08	[69]
Golden pompano (*Trachinotus blochii*)	Hypoxia challenge test	Acute	24 h	t_LOE_ (min)	BSR-seq	SNP calling from RNA-seq	821,398	42	16 QTL	NA	NA	[96]
Blunt snout bream (*Megalobrama amblycephala*)	Hypoxia challenge test	Acute	NA	LOE_crit_ (mg/L)	Whole-genomic mutagenesis to increase hypoxia tolerance	Whole-genome resequencing	3,195,434	6	NA	NA	NA	[97]

NA: not available; Fish: Number of genotyped fish; QTLs: number of significant (a) or suggestive (b) marker associated with hypoxia tolerance trait(s); PVE: Phenotypic variance explained of the most significant marker; *h*^2^: heritability

**Table 4:** Number of candidate genes proposed (n) and signaling pathways and cellular processes involved for hypoxia tolerance

Species	*n*	Main candidate genes identified	Signaling pathways and Cellular Processes involved	Reference
Nile tilapia (*Oreochromis niloticus*)	1	HIF1αn (also known as FIH)	HIF-1 signaling pathway	[91]
*Barramundi* (*Lates calcarifer*)	1	HIF1αn (also known as FIH)	HIF-1 signaling pathway	[92]
Channel catfish (*Ictalurus punctatus*)	15	*lrrcl*, *tceb3*, *mlip*, *fam83b*, *gclc*, *fgfr2*, *plpp4*, *fbxo9*, *bmp5*, *bag2*, *nf1*, *lgals9*, *ucp2*, *gdnf*, *dhrs13*	MAPK pathway, PI3K/AKT/mTOR signaling pathway, hypoxia-mediated angiogenesis, cellular proliferation, apoptosis, survival	[70]
♂ Hybrid F × ♀ Channel catfish Hybrid F1: ♀ Channel catfish × ♂ Blue catfish (*I. punctatus* × *I. furcatus*)	125	*dmbx1a*, *pif1*, *ptger4*, *artn*, *st3gal3a*, *kdm4a*, *ptprf*, *pkib*, *cyp1a1*, *ccbe1*, *sema7a*, *arid3a*, *arid3b*, *fam219b*, *p2ry1*, *rap2b*, *klhl5*, *fam114a1*, *klf3*	VEGF pathway, MAPK pathway, PI3K/AKT/mTOR signaling pathway, p53-mediated apoptosis, damage checkpoint	[49]
Large yellow croaker (*Larimichthys crocea*)	12	*mybpc1*, *atp1a1*, *prmt5*, *psmb5*, mybpc1, atp1a1, *egln2*, *pygm*, *camk2d*, *arsj*, *trmt10a*, *aco1*	HIF signaling pathway, oxidative stress, energy metabolism, ion regulation	[64]
Large yellow croaker (*Larimichthys crocea*)	22	*pds5a*, *smin14*, *ugdh*, *lias*, *rfc1*, *klf3*, *tbc1d1*, *pgm2*, *melk*, *thsd4*, *polq*, *camk2d2*, *ankb*, *pgd*, *wfs1a*, *gpi*, *stim2a*, *slc34a2a*, *ho1*, *nfix*, *ccna2*, *grik4*	DNA replication and repair, glucose metabolism, erythropoiesis, glucose transport, iron metabolism, ion regulation, pentose phosphate pathway	[5]
Rainbow trout (*Oncorhynchus mykiss*)	15	*ids*, *fmr1*, *arx*, *lonrf3*, *commd5*, *map4k4*, *smu1*, *b4galt1*, *re1*, *abca1*, *noa1*, *igfbp7*, *noxo1*, *bcl2a*, *mylk3*	Glycogen metabolism, glucose metabolism, cation regulation, DNA repair, HIF-1α regulation, mitochondrial metabolism, hematopoiesis, angiogenesis, oxidative response pathways, apoptosis	[65]
Nile tilapia (*Oreochromis niloticus*)	2	*gpr132*, *abcg4*	Lactate sensing and signaling, oxidative stress protection	[91]
Pompano (*Trachinotus ovatus*)	16	*lonrf3*, *commd5*, *fam199x*, *gpr137*, *pld7*, *syvn1*, *smad5*, *mdga1*, *gabra4*, *ap1ar*, *cfap100*, *pold1*, *zgc:55,558*, *trit1*, *kif18a*, *NEK3*	glycolysis, DNA repair, acid balance, apoptosis	[63]
Golden pompano (*Trachinotus blochii*)	1116	*PTGS2*, *CYLD*, *Ifih1*	Anaerobic metabolism, stress response, immune response, waste discharge, cell death	[96]
*Pelteobagrus vachelli*	1	*sema7a*	Immune processes	[94]
Silver sillago (*Sillago sihama*)	7	*cyp20a1*, *mgst3b*, *kcnh2*, *cluh*, *adk*, *xdh*, *slc19a2*	Xenobiotic biodegradation, defense against oxidative stress, membrane potential equilibrium, mitochondrial integrity, ribose metabolism, nucleotide metabolism, ion transport and caption	[95]
Blunt snout bream (*Megalobrama amblycephala*)	1	*egln2*	HIF-1 signaling pathway	[67]
Blunt snout bream (*Megalobrama amblycephala*)	1	*hif2αb*	HIF-1 signaling pathway	[93]
Blunt snout bream (*Megalobrama amblycephala*)	4	*Epo X1*, *VEGFR1*, *HO-1a*, *LPAR6*	HIF-1 signaling pathway, VEGF pathway, FOXO, Janus kinases (JAKs), signal transducer and activator of transcription proteins (STATs), MAPK Pathway, PI3K/AKT/mTOR signaling pathway	[107]

### Overview of key results from study selection and data extraction

**Fig. 1:**
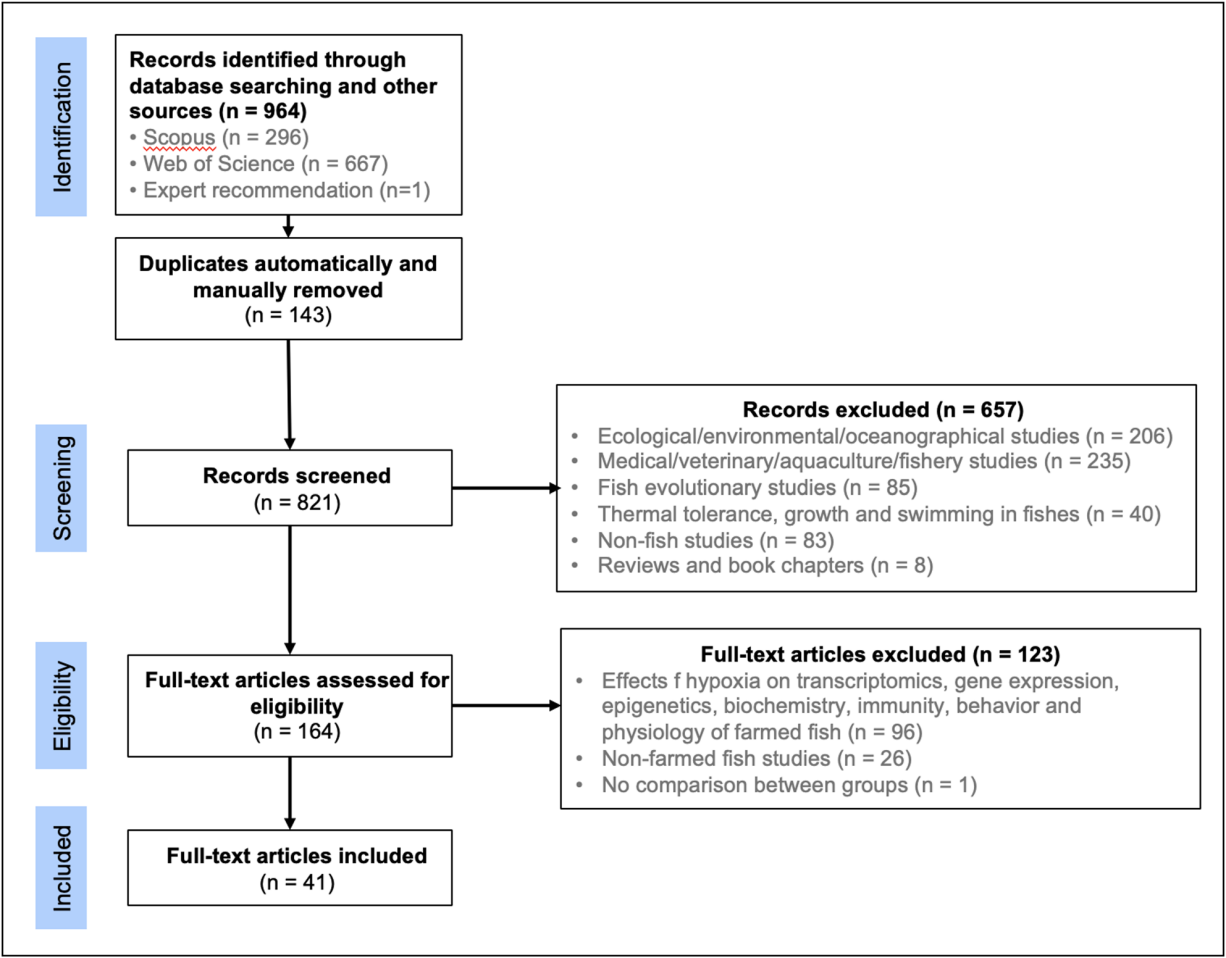
PRISMA flow diagram showing the process of filtering and selecting articles for this systematic review

Our search strategy across two databases retrieved 963 articles. In addition, one article was incorporated following the recommendation of an anonymous expert, bringing the total to 964 articles. After removing 143 duplicates and excluding 779 articles that did not meet the inclusion criteria, 41 studies were selected for analysis (Fig. 1). In total, the review identified studies on hypoxia tolerance in 26 farmed fish species and three hybrid lines, including blunt snout bream (*Megalobrama amblycephala*), rainbow trout (*Oncorhynchus mykiss*), common carp (*Cyprinus carpio*), Nile tilapia (*Oreochromis niloticus*), bighead carp (*Aristichthys nobilis*), grass carp (*Ctenopharyngodon idellus*), Atlantic salmon (*Salmo salar*), channel catfish (*Ictalurus punctatus*), among others. The selected studies were categorized into two groups: (a) traditional genetics and selective breeding studies (*n* = 25), which assessed hypoxia tolerance at the phenotypic level by comparing strains, genetic lines, families, or hybrids (Table 2), and (b) modern genomics and biotechnology studies (*n* = 16), which investigated hypoxia tolerance using advanced genomic approaches such as GWAS, GS, or mutagenesis (Table 3). In addition, candidate genes related to hypoxia response mechanisms identified in the reviewed articles were summarized (Table 4). Accordingly, the main trends emerging from each table are described in the following paragraphs.

Most phenotypic studies (Table 2) evaluated hypoxia tolerance using scalable challenge tests such as time to loss of equilibrium or dissolved oxygen at LOE. Other less frequently used approaches included respirometry-based P_crit_, survival time (min) and survival status (alive or dead) associated with mortality-based hypoxia tests, as well as behavioral indicators such as ASR. Only a handful of species accounted for the majority of studies—particularly rainbow trout, Nile tilapia, blunt snout bream, and common carp—while comparisons spanned strains, families, hybrids, ploidy (diploid vs. triploid), and life stages. This diversity highlights substantial within- and among-species variation but also underscores the challenges of comparing hypoxia tolerance phenotypes, given the heterogeneous endpoints and measurement units (minutes, mg/L, kPa, percent air saturation, alive or dead).

In parallel with the phenotypic heterogeneity described in Table 2, modern genomics studies (Table 3) applied diverse approaches ranging from SNP association and GWAS to QTL mapping, GS, and mutagenesis. Sample sizes and marker densities varied widely, complicating cross-study comparisons. Nevertheless, two consistent patterns emerged: (1) the most significant loci generally accounted for only a modest proportion of phenotypic variance (typically < 25–30%), and (2) only a small fraction of studies reported heritability estimates. When available, heritability estimates ranged from moderate (0.26 in grass carp) to high (0.61 to 0.65) in large yellow croaker.

Candidate gene studies (Table 4) revealed that different genetic and regulatory mechanisms can underlie hypoxia tolerance. Genes and markers associated with hypoxia tolerance were often linked to hypoxia signaling and its regulation (HIF-related genes and their inhibitors, such as *HIF1αn*), angiogenesis (e.g., VEGF), energy metabolism (glycolysis, PI3K/AKT/mTOR), oxidative stress response, erythropoiesis, ion regulation, immune response or apoptosis.

### Methods and traits for assessing hypoxia tolerance

**Fig. 2:**
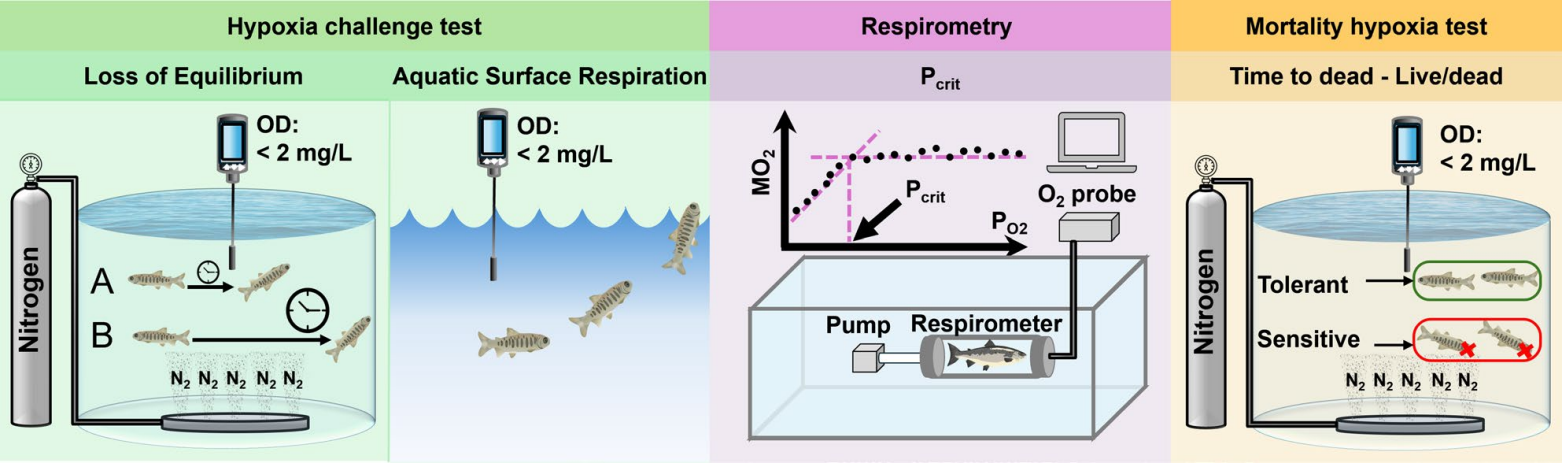
Methods to assess hypoxia tolerance in fish. The hypoxia challenge test quantifies tolerance using tLOE/LOEcrit, the point at which fish lose equilibrium, or by measuring ASR as fish access the oxygen-rich surface layer under low dissolved oxygen (OD < 2 mg/L). Respirometry estimates Pcrit by recording oxygen consumption as PO₂ declines. The mortality hypoxia test evaluates time to death and classifies fish as tolerant (live) or sensitive (dead) under hypoxic conditions

Since accurate and cost-effective phenotyping is essential for implementing selective breeding programs in aquaculture, it is relevant to first discuss the methods and specific traits used to assess hypoxia tolerance. A total of 25 traditional genetics and selective breeding studies were retrieved by our search strategy (Fig. 1; Table 2). In these, hypoxia tolerance was primarily assessed using three approaches (Fig. 2): (1) hypoxia challenge tests (measuring t_LOE_ or LOE_crit_), (2) mortality hypoxia tests (assessing survival time or status), and (3) respirometry (measuring P_crit_). Less commonly used indicators included ASR and aerial emergence, sometimes referred to as “floating head”. ASR describes the selective uptake of oxygen-rich water from the surface layer, while aerial emergence involves leaving the water to breathe air directly.

The LOE approach has been widely used in genetic studies of hypoxia tolerance because of its simplicity, speed, and minimal equipment requirements, enabling high-throughput screening in a short time (Table 2) [98]. However, observer bias can influence results, requiring standardized training for personnel conducting the assessments. In contrast, the mortality hypoxia test, based on survival time or status (alive or dead), provides an observable endpoint that can be used to identify individuals with extreme hypoxia tolerance, complementing information obtained from LOE measurements (Table 2). However, ethical concerns arise due to the use of lethal hypoxia levels, particularly in large-scale trials involving hundreds or thousands of fish. A common limitation of the aforementioned methods is that they assess acute hypoxia responses rather than long-term adaptation to low-oxygen environments. By contrast, P_crit_ measured via respirometry provides detailed physiological insight into oxygen uptake efficiency under hypoxic conditions. Additionally, P_crit_ is non-lethal, allowing for repeated measurements on the same individuals, similar to hypoxia challenges using LOE as an endpoint [60]. Despite these advantages, respirometry is complex, expensive, and time-consuming, which restricts its feasibility in large-scale breeding programs. Based on our evaluation of the studies included in this review, loss of equilibrium–based traits (t_LOE_ or LOE_crit_) and survival-related outcomes (time-to-death or live/dead status) emerge as the most practical and scalable phenotypes for routine selection in breeding programs. In contrast, indicators linked to metabolic capacity (e.g., P_crit_) or some behavioral responses to hypoxia such as the ASR are less suited for routine application in breeding at an operational scale.

### Association of hypoxia tolerance with other economically relevant traits

Effective breeding for hypoxia tolerance requires knowing how it genetically correlates with key traits such as growth, thermal tolerance and disease resistance. These correlations may be positive or negative, affecting the practicality of breeding programs. However, in the present systematic review, no studies were identified that reported formal estimates of genetic correlations between hypoxia tolerance and other traits. Nevertheless, several studies reported phenotypic correlations or provided indirect comparative evidence, indicating species-specific associations between hypoxia tolerance and these economically relevant traits.

For instance, the genetically improved farmed tilapia (GIFT) strain, known for its fast growth, was compared with the native, slower-growing Akosombo strain to evaluate differences in hypoxia tolerance [73]. The GIFT strain exhibited a significantly higher oxygen consumption rate (MO_2_), engaged in ASR at higher oxygen levels, and showed an increased ventilatory frequency (f_v_) compared to the Akosombo strain, suggesting lower hypoxia tolerance in the GIFT strain. However, this negative association between growth rate and hypoxia tolerance is not always observed. In a study on common carp (*Cyprinus carpio*), researchers compared survival under low dissolved oxygen between a normal strain and a transgenic F_2_ strain carrying a trout growth hormone (rtGH) transgene [87]. Interestingly, the transgenic carp exhibited a longer mean survival time under hypoxic conditions, suggesting a beneficial pleiotropic effect of the inserted transgene on hypoxia tolerance. Similarly, a study in rainbow trout (*Oncorhynchus mykiss*) compared two strains with differing growth rates for their t_LOE_ under hypoxia [66]. Significant inter-individual variation was observed, but overall, the fast-growing strain demonstrated greater hypoxia tolerance (t_LOE_: 180–410 min) compared to the slow-growing strain (t_LOE_: 130–280 min).

Thermal resistance is another key trait linked to hypoxia tolerance. Thermal tolerance is a heritable trait, with heritability estimates ranging from 0.26 for upper thermal tolerance (UTT) in grass carp (*Ctenopharyngodon idella*) [99] to 0.40 for critical thermal maximum (CTmax) in Atlantic salmon (*Salmo salar*) [100]. The concept of oxygen-capacity-limited thermal tolerance (OCLTT) suggests a strong relationship between these two physiological limits; however, the strength and direction of this relationship appear to be species dependent [101]. In Nile tilapia, a negative phenotypic correlation (*r* = − 0.76) was estimated between hypoxia tolerance measured as ASR and thermal tolerance measured as CTmax [74]. In contrast, in Atlantic salmon, the estimate of the phenotypic correlation between thermal tolerance (CTmax) and hypoxia tolerance (t_LOE_) was positive (Spearman’s correlation, ρ = 0.69) (63). More recently, Lagarde et al. [102], using six isogenic rainbow trout lines, found no overall relationship between acute hyperthermia tolerance and hypoxia tolerance. While some lines were resistant to both stressors, others were resistant to only one but sensitive to the other, indicating the absence of strong antagonistic genetic effects between these traits. These contrasting patterns highlight that the correlation between thermal and hypoxia tolerance varies across species, with important implications for selective breeding strategies. Strong negative genetic correlations may limit the simultaneous improvement of both traits in a breeding program, whereas the absence or presence of a positive genetic correlation would facilitate their joint selection. Future studies that combine both stressors will be key to identify genotypes resilient to multifactorial climate challenges.

In contrast to growth and thermal tolerance, the relationship between hypoxia tolerance and pathogen resistance remains poorly studied; in this systematic review, only a single study addressing this relationship was identified, and it was conducted in rainbow trout [88]. Fetherman et al. [88], compared hypoxia tolerance (measured as LOE) between a rainbow trout strain resistant to Myxobolus cerebralis, the causative agent of whirling disease, and a non-resistant strain. No significant differences in hypoxia tolerance were detected between the two groups [88].

### Variation in hypoxia tolerance across strains and populations

Several studies emphasized the need to evaluate hypoxia tolerance across strains and populations, as understanding inter-strain and inter-population variation is essential for optimizing breeding programs that enhance resilience to low-oxygen environments (Table 2). Accordingly, differences in hypoxia tolerance have been studied among strains of rainbow trout, Atlantic salmon, Nile tilapia and channel catfish (Table 2). For example, significant differences in hypoxia tolerance have been studied among strains of rainbow trout [61]. Similarly, a study on Atlantic salmon (*Salmo salar*) assessed embryo survival under hypoxia across four genetically distinct populations from different rivers in France, revealing significant differences among them [75]. In channel catfish (*Ictalurus punctatus*), a broad range of hypoxia tolerance has also been reported, with t_LOE_ ranging from 8 to 104 min across six different strains [77]. These findings suggest a strong genetic component underlying hypoxia tolerance, potentially influenced by local adaptation or historical selection pressures. However, not all species exhibit clear interpopulation differences. A study on barramundi (*Lates calcarifer*) compared two geographically distinct populations—one from a tropical river with strong oxygen fluctuations and another from a subtropical river—and found no significant interpopulation differences in P_crit_ [76]. The authors suggested that in this species, physiological plasticity may play a more dominant role than local genetic adaptation in determining hypoxia tolerance [76]. Importantly, as shown by this review, the absence of detectable interpopulation differences does not preclude substantial genetic variation among individuals and families within the same population or strain. Overall, these findings support a key role of genetic variation in hypoxia tolerance and highlight the relevance of both between-strain comparisons and within-strain (or within-population) selection for improving performance under low-oxygen conditions.

### Effects of ploidy and gynogenesis on hypoxia tolerance

Other studies identified by our search examined the effects of triploid and gynogenetic strains on hypoxia tolerance in fish (Table 2). Triploid strains, for example, generally exhibited lower or slightly reduced hypoxia tolerance compared to diploid strains. In rainbow trout, triploid (3n) strains exhibited lower hypoxia tolerance than diploid (2n) strains [82], primarily due to reduced gill surface area. Similarly, in brook trout (*Salvelinus fontinalis*), triploids (3n) were less tolerant to hypoxia than diploids (2n), although the difference was very small [84]. However, another study found that when diploid and triploid rainbow trout were exposed for 1 h to temperatures above their thermal optima, no significant effect of ploidy was found on hypoxia tolerance [83]. In the context of gynogenetic strains, enhanced hypoxia tolerance has been reported in blunt snout bream (*Megalobrama amblycephala*) [85]. Two gynogenetic lines, generated by activating eggs with UV-irradiated sperm followed by a cold shock, exhibited greater hypoxia tolerance than the normal strain, although the specific mechanisms underlying this improvement remain unclear [85]. Likewise, another study generated a gynogenetic strain by activating eggs with UV-inactivated red crucian carp spermatozoa, producing individuals with superior hypoxia tolerance compared to the control group [86]. Although the number of available studies remains limited, these findings suggest that gynogenesis may represent a more suitable strategy than triploidy for developing hypoxia-tolerant lines in aquaculture. However, further research is needed to elucidate the genetic and physiological mechanisms driving these improvements and to assess their practical applications in selective breeding programs.

### Hybridization and heterosis as enhancers of hypoxia tolerance

Hybridization is a well-known method for improving valuable aquaculture traits, including hypoxia tolerance (Table 2). By crossing strains from the same or closely related species, studies have explored whether hybrid vigor (heterosis) can improve resilience to low-oxygen conditions. For instance, a study comparing the hypoxia tolerance of blunt snout bream (*Megalobrama ampblycephala*, BSB) and its hybrid (M. *amblycephala* ♀ × *Culter alburnus* ♂, BTBB) found that the hybrid exhibited significantly greater tolerance. The BTBB hybrid had a lower mean LOE_crit_ (0.5 ± 0.01 mg/L) and fewer individuals losing equilibrium (5.3 ± 0.6 fish) compared to BSB (0.9 ± 0.03 mg/L, 24.7 ± 1.5 fish), suggesting strong heterosis (79). A separate study by Chen et al. [103] reinforced these findings, reporting that hybrids of *M. amblycephala* × *Culter alburnus* inherited broad hypoxia tolerance from *C. alburnus*. Similarly, artificial hybridization between *Pelteobagrus fulvidraco* and *Leiocassis longirostris* produced two hybrid lines: PL (*P. fulvidraco* ♀ *× L. longirostris* ♂) and LP (*L. longirostris* ♀ × *P. fulvidraco* ♂) [89]. The PL hybrid exhibited a higher hatching rate, expected morphological traits, and greater hypoxia tolerance, as evidenced by increased enzyme activity and upregulation of HIF-related genes [89]. In contrast, hybridization outcomes in catfish appear more variable. In catfish, hybrids of *Ictalurus punctatus* × *Ictalurus furcatus* displayed varying degrees of hypoxia tolerance depending on the geographical origin of the *I. furcatus* parent strain, suggesting that local thermal regimes influence this trait [90]. Lastly, hybridization has also been explored in disease-resistant strains. A cross between resistant and non-resistant *Oncorhynchus mykiss* strains for *Myxobolus cerebralis* exhibited greater hypoxia tolerance than one of the pure lines [88]. Overall, these studies underscore the potential of hybridization as a viable strategy to enhance hypoxia tolerance in aquaculture species, leveraging both hybrid vigor and genetic contributions from specific parental lineages to develop more resilient fish strains.

### Interspecific variation in hypoxia tolerance

Hypoxia tolerance varies not only within species but also across aquaculture-relevant species, influencing their suitability for specific culture conditions. Several studies have explored these interspecific differences (Table 2), providing valuable insights for aquaculture management. In China, a study evaluating six commercially important cyprinid species from the Yangtze River found significant variation in their P_crit_, indicating that each species has different oxygen demands and culture requirements [80]. Similarly, another study [50] assessed hypoxia tolerance in nine cyprinid species, including two strains of *Cyprinus carpio*. The results showed a broad range of hypoxia tolerances: *Carassius carassius* (Crucian carp) and *Carassius auratus* (goldfish, not farmed species), both adapted to slow-moving water bodies, displayed extreme tolerance (LOE_crit_ ~ 0 kPa). Six species exhibited moderate tolerance (LOE_crit_: 0.1–0.3 kPa), whereas thick-jawed bream (*Megalobrama pellegrini*) and qingbo (*Spinibarbus sinensis*), which typically inhabit fast-flowing rivers, were the most sensitive (LOE_crit_ ~ 0.6 kPa). Interestingly, hypoxia tolerance (LOE_crit_) did not correlate with P_crit_, suggesting that P_crit_, often considered a standard metric for evaluating hypoxia tolerance, may not always serve as a reliable indicator on its own, and that multiple metrics should be employed [50]. A follow-up study by Fu et al. [51] expanded this analysis to 12 cyprinid species from habitats with different flow regimes (rapid, slow, and intermediate). Consistent with previous findings, species from fast-flowing environments exhibited lower hypoxia tolerance than those from slow-moving waters, further supporting the idea that hypoxia tolerance is more strongly influenced by habitat than by phylogenetic lineage [51]. Beyond cyprinids, hypoxia tolerance has also been studied in *Anoplopoma fimbria*, a species gaining interest in aquaculture. A study comparing juveniles and adults found that adults, which naturally inhabit deep oxygen minimum zones (~ 1500 m), exhibited significantly higher hypoxia tolerance (LOE_crit_: ~5.4% oxygen saturation) than juveniles (LOE_crit_: ~8.3% oxygen saturation) [81]. These findings underscore the complexity of hypoxia tolerance across species, highlighting the importance of considering ecological adaptations and life stage differences when developing aquaculture strategies.

### Modern genetics and selective breeding studies of hypoxia tolerance

Recent advances in genetics and genomics have significantly contributed to the understanding of hypoxia tolerance in farmed fish. In this systematic review, a total of 16 modern genetic studies were identified. They employed diverse methodologies to characterize the genetic architecture of hypoxia tolerance (Table 3), including SNP association studies (*n* = 2) [92, 104], haplotype or diplotype association studies (*n* = 2) [67, 93], GWAS (*n* = 6) [5, 49, 63–65, 70], GS (*n* = 2) [65, 69], QTL mapping (*n* = 3) [91, 94, 95], bulked segregant RNA-Seq (*n* = 1) [96], and whole-genomic mutagenesis (*n* = 1) [97]. In most cases, hypoxia tolerance was assessed through LOE experiments. However, the classification criteria for hypoxia-sensitive (HS) and hypoxia-tolerant (HT) individuals varied widely among studies. For instance, some studies defined HS individuals as the first 10% to lose equilibrium and HT individuals as the last 10% [63, 64, 91], whereas others applied alternative thresholds, including the first and last 5% [5], 17.5% [96], or 35% [70] of individuals. Despite these methodological differences, all these studies identified QTL, SNPs, potential candidate genes, and the biological pathways in which these genes might be involved (Table 4). These findings underscore the genetic basis of hypoxia tolerance and provide valuable insights for selective breeding programs aimed at improving resilience to low-oxygen environments in aquaculture.

### Analysis of association between single genes and hypoxia tolerance

Two studies identified in our search examined the genetic basis of hypoxia tolerance by analyzing single associations between SNPs in key genes and hypoxia-related traits. Li et al. [104] identified a significant association of a SNP (8892953 G > A) located in the fourth intron of the hypoxia-inducible factor inhibitor gene (*HIF1αn*) with hypoxia tolerance in Nile tilapia (*Oreochromis niloticus*) [104]. To reduce ambiguity in classifying intermediate phenotypes, only the 192 fish with extreme t_LOE_ values (most sensitive and most tolerant) were genotyped. Fish with the A/A genotype (*n* = 77) exhibited the longest t_LOE_ under hypoxic stress (252.3 min, *n* = 87), followed by A/G heterozygotes (194.3 min, *n* = 87), and G/G homozygotes (191.6 min, *n* = 17) [104]. Similarly, a study on Barramundi (*L. calcarifer*) identified three SNPs in the third and fourth introns of *HIF1αn*, with SNP 9,332,241 (C/T) in the fourth intron being significantly associated with hypoxia tolerance; specifically, the C-allele frequency was 18.2% in surviving fish and 8.9% in hypoxia-dead fish [92]. Although limited in number, these studies highlight the potential relevance of single-gene variation in *HIF1αn* for hypoxia tolerance in farmed fish.

### GWAS and QTL mapping

GWAS and QTL mapping are powerful approaches for identifying genetic variants linked to hypoxia tolerance (Table 3). These approaches have been applied in multiple farmed fish species, revealing a complex genetic architecture underlying the different traits used to measure hypoxia tolerance.

In channel catfish (*Ictalurus punctatus*), Wang et al. [70] employed both “within-strain” and “between-strain” approaches to identify significant QTL and SNPs associated with hypoxia tolerance [70]. In the within-strain analysis, the Kansas strain showed 26 significant SNPs, while the Kmix and Thompson strains exhibited four and one significant SNP, respectively [70]. The most significant SNPs in each strain accounted for high percentages of phenotypic variation (25.3, 23.0, and 32.0%, respectively). In the remaining three strains (103KS, Marion, and MarionS), no SNPs were significantly associated with hypoxia tolerance, although some were showing suggestive associations [70]. In the across-strain analysis, only one significant SNP was detected, which explained 5.7% of phenotypic variance [70]. Given that the SNPs identified in the strain-specific analyses appeared more important and numerous than those detected in the combined analysis, the authors suggest that strain-by-strain GWAS analysis is the preferred approach for identifying SNPs/QTL for breeding purposes [70]. Another GWAS examining hypoxia tolerance in channel catfish × blue catfish [49] hybrid detected significant SNPs that differed from those identified in the six pure channel catfish strains previously studied by Wang et al. [70]. The authors attributed this discrepancy to the complex genetic architecture of the trait, as well as to differences in genetic background, phenotypic variance explained by individual loci, sample size, and the number of families included in the analysis [49], all of which can substantially affect the power and consistency of GWAS results.

In Rainbow trout, a GWAS on hypoxia tolerance identified three significant QTL: two on chromosome 31 and one on chromosome 20 [65]. Additionally, two putative QTL with suggestively associated SNPs were found, one located on chromosome 15 and the other on chromosome 28, each with a single SNP. The most significant SNPs from each QTL explained between 0.2 and 0.8% of the genetic variance. The authors note that, unlike other species where the most significant (“peak”) SNPs explained a large percentage of phenotypic variance (Table 3), in rainbow trout these SNPs had minimal effects, reinforcing the idea that hypoxia tolerance in this species has a highly polygenic nature, governed by multiple loci with small individual effects [65]. Further, the authors proposed that one of the QTL identified on chromosome 31 may correspond to a supergene [105], based on the presence of six functionally relevant candidate genes clustered within the QTL region and relatively high linkage disequilibrium (r² = 0.45) compared with genome-wide averages. According to this hypothesis, tightly linked neighboring genes could segregate together and jointly contribute to the complex physiological, morphological, and behavioral responses to acute hypoxia in rainbow trout.

Large yellow croaker (*Larimichthys crocea*) is one of the most economically important marine aquaculture species in East Asia, particularly in China, which has motivated increasing interest in improving its tolerance to hypoxic conditions through genetic and genomic approaches. In large yellow croaker (*Larimichthys crocea*), Ding et al. [64] used *de novo* SNP genotyping by ddRAD-Seq (Double-digest restriction-site associated DNA sequencing) identifying two significant SNPs for survival time and four for status of survival. Another study on the same species detected seven suggestively significant SNPs based on GWAS and several candidate genes based on GWAS and transcriptomic analysis [5]. A recent GS study on hypoxia tolerance in large yellow croaker, phenotyped 753 individuals for time survival (hours) and survival status (binary trait: hypoxia-sensitive HS, hypoxia-tolerant HT), and genotyped them using 38,472 high-quality SNPs [69]. Substantial phenotypic variation was observed in this population, with survival time ranged from 20.13 to 38.6 h, yielding high heritability estimates of 0.62 for survival time and of 0.65 for survival status [69], supporting the feasibility of genomic approaches to improve hypoxia tolerance in this species.

Other, less-studied aquaculture species further illustrate the heterogeneous genetic architecture underlying hypoxia tolerance. In golden pompano (*Trachinotus ovatus*), a whole-genome sequencing-based study identified four significant SNPs associated with hypoxia tolerance [63]. Similarly, in darkbarbel catfish (*Pelteobagrus vachelli*), ddRAD-Seq-based linkage mapping enabled the identification of a single significant QTL and one putative candidate gene associated with hypoxia tolerance (Table 4) [94]. In Nile tilapia, multiple genomic regions associated with hypoxia tolerance have also been reported, including four highly significant QTL and two candidate genes (Table 4) [91]. Likewise, in silver sillago (*Sillago sihama*), QTL mapping revealed six QTLs associated with hypoxia tolerance and seven candidate genes. Among these, *mgst3b* emerged as a particularly promising candidate, as it is a member of the glutathione S-transferase (GST) gene family, which could play an important role in cellular defense against oxidative stress and detoxification under hypoxic stress conditions [95].

### Bulked segregant analysis: RNA seq (BSR-Seq)

A technique known as BSR-Seq analysis, which combines RNA-Seq with Bulked Segregant Analysis (BSA) to correlate global gene expression patterns with SNPs [96], was applied in golden pompano (*Trachinotus blochii).* The authors found that hypoxia tolerance-related SNPs in this fish were mainly associated with the linkage groups 18 and 22. Numerous candidate genes were proposed (Table 4), which are involved in anaerobic energy metabolism, stress response, immune response, waste discharge, and cell death. These findings highlight the utility of BSR-Seq in identifying genetic variants and molecular mechanisms underlying hypoxia tolerance, providing potential markers for genetic selection in aquaculture.

### Selective breeding results

Although interest in improving hypoxia tolerance through selective breeding has increased, only one study identified in this review has successfully implemented a multi-generational breeding program [53]. This selective breeding initiative, conducted at the Bream Genetics and Breeding Center (BGBC) at Shanghai Ocean University, was based on a wild-derived population of *Megalobrama amblycephala* from Poyang Lake and involved successive generations selected for enhanced hypoxia tolerance. The resulting F4 generation exhibited significantly lower loss-of-equilibrium thresholds (LOEcrit) than the control group at 10, 25, and 30 °C, indicating a consistent improvement in hypoxia tolerance across different thermal conditions [53]. In the F5 generation, exonic SNPs in the *egln2* gene were associated with enhanced hypoxia tolerance, as evidenced by increased erythrocyte and hemoglobin levels under hypoxic conditions, elevated catalase and superoxide dismutase activity, lower LOEcrit values, and reduced gill lamellar remodeling compared with other diplotypes [67]. More recently, in a newly developed variety of the same species, designated ‘Pujiang No. 2’, the *hif2αb* gene was investigated for its association with hypoxia tolerance after four successive generations of selection, using wild fish as a reference population [93]. One diplotype was associated with enhanced hypoxia tolerance, as evidenced by lower LOEcrit values, higher erythrocyte counts, and increased catalase and superoxide dismutase activity compared with other diplotypes [93].

### Gene editing and hypoxia tolerance mutant lines

Precise genomic modification using gene editing techniques and other mutagenesis approaches presents new opportunities for developing hypoxia tolerant fish. For example, gene editing has been applied to blunt snout bream (*Megalobrama amblycephala*) to create knockout mutants of the erythropoietin (EPO) gene [106]. EPO is a glycoprotein hormone that plays a key role in regulating erythropoiesis and is a classic hypoxia-responsive gene in the HIF-1 pathway. Interestingly, EPO^−/−^ mutants exhibited reduced red blood cell counts, lower hemoglobin levels, and increased oxygen tension thresholds under hypoxia, highlighting the crucial role of EPO in hypoxia tolerance in this species [106].

There is also evidence that it is feasible to develop hypoxia-tolerant fish using techniques such as Atmospheric and Room Temperature Plasma (ARTP) mutagenesis. For example, in blunt snout bream (*Megalobrama amblycephala*), ARTP mutagenesis was used to generate complete genomic mutants [97]. By applying ARTP to semen from a gynogenetic male, 2,026 mutant offspring were produced, of which 384 showed improved critical oxygen levels (LOE_crit_: 0.45 mg/L) compared to the control group (LOE_crit_: 0.86 mg/L) after three months of culture. Genome resequencing revealed 3,651 nonsynonymous mutations across 1,223 genes in ARTP mutants, with four genes (*Epo X1*, *VEGFR1*, *HO-1a*, *LPAR6*) showing differential expression under hypoxia. These four genes are involved in key pathways such as HIF-1, VEGF, FOXO (Forkhead box O), JAK-STAT (Janus kinase/Signal Transducer and Activator of Transcription), MAPK, mTOR, and PI3K-Akt [106]. Based on these results, the authors proposed using these ARTP-induced mutations as potential markers for selective breeding to enhance hypoxia tolerance in blunt snout bream [97].

## Conclusions

Overall, hypoxia tolerance, defined operationally through multiple phenotypic proxies, appears to be an inherited polygenic trait characterized by a complex genetic architecture, in which variation is governed by multiple polymorphisms distributed throughout the genome, each exerting small to moderate effects. The wide phenotypic variability observed in hypoxia tolerance among farmed fish is largely attributed to the high genetic diversity within and among populations. Importantly, evidence from multiple species suggests that different genetic and regulatory mechanisms can underlie hypoxia tolerance, rather than implying a shared or uniform molecular basis across taxa. Consequently, the identification of species-specific QTLs, SNPs, and candidate genes remains essential. Integrating GWAS with transcriptome analyses enhances detection power, while the use of large sample sizes and medium to high density SNP microarrays significantly increases the likelihood of identifying statistically significant markers and candidate genes. These advancements will facilitate the development of genetically improved fish strains with enhanced resilience to hypoxic stress. Furthermore, laboratory based genetic modifications, such as gynogenesis, transgenesis, and potentially gene editing, offer promising avenues for developing desired phenotypic traits.

On the other hand, development of an optimal breeding strategy—whether GS or marker assisted selection—depends on the proportion of phenotypic variance explained by significant SNPs. In species such as rainbow trout and yellow croaker, where hypoxia tolerance is highly polygenic and SNPs exhibit small individual effects, GS is the preferred approach. Conversely, in species like channel catfish and golden pompano, where specific SNPs explain a larger proportion of phenotypic variance, marker assisted selection may be a viable strategy. These findings underscore the necessity of understanding the genetic architecture of hypoxia tolerance in each species to implement the most effective selective breeding programs.

At the same time, some practical and biological constraints must be considered. From a phenotyping perspective, not all hypoxia-related traits are equally scalable for large breeding programs: loss-of-equilibrium–based measures (e.g. time to LOE) and survival time or survival status are more amenable to high-throughput application than physiologically intensive traits such as critical oxygen tension (Pcrit) or aquatic surface respiration (ASR). Moreover, genetic correlations between hypoxia tolerance and other economically important traits—such as growth, disease resistance, and thermal tolerance—remain largely unknown. Future research should quantify these correlations to guide multi trait selection strategies. Commercial deployment of genomic technologies faces challenges related to regulatory frameworks and societal concerns about gene edited or transgenic fish, while the costs of high throughput genotyping may be particularly limiting in developing countries.

## Data Availability

Not applicable.
